# A new perspective on positive symptoms: expression of damage or self-defence mechanism of the brain?

**DOI:** 10.1007/s10072-024-07395-x

**Published:** 2024-02-14

**Authors:** Annibale Antonioni, Emanuela Maria Raho, Mariachiara Sensi, Francesco Di Lorenzo, Luciano Fadiga, Giacomo Koch

**Affiliations:** 1 Doctoral Program in Translational Neurosciences and Neurotechnologies, Department of Neuroscience and Rehabilitation, University of Ferrara, Via Ludovico Ariosto 35, 44121 Ferrara, Italy; 2 Department of Neuroscience and Rehabilitation, University Unit of Neurology, University of Ferrara, 44121 Ferrara, Italy; 3 Unit of Neurology, Interdistrict Health Care Department of Neuroscience, S. Anna Ferrara University Hospital, 44124 Ferrara, Italy; 4 Non Invasive Brain Stimulation Unit, Istituto Di Ricovero E Cura a Carattere Scientifico Santa Lucia, 00179 Rome, Italy; 5 Center for Translational Neurophysiology, Istituto Italiano Di Tecnologia, 44121 Ferrara, Italy; 6 Section of Physiology, Department of Neuroscience and Rehabilitation, University of Ferrara, 44121 Ferrara, Italy

**Keywords:** Positive neurological symptoms, Dopamine, Neuroplasticity, Active inference theory, Defensive activation theory

## Abstract

Usually, positive neurological symptoms are considered as the consequence of a mere, afinalistic and abnormal increase in function of specific brain areas. However, according to the Theory of Active Inference, which argues that action and perception constitute a loop that updates expectations according to a Bayesian model, the brain is rather an explorer that formulates hypotheses and tests them to assess the correspondence between internal models and reality. Moreover, the cerebral cortex is characterised by a continuous “conflict” between different brain areas, which constantly attempt to expand in order to acquire more of the limited available computational resources, by means of their dopamine-induced neuroplasticity. Thus, it has recently been suggested that dreams, during rapid eye movement sleep (REMS), protect visual brain areas (deprived of their stimuli during rest) from being conquered by other normally stimulated ones. It is therefore conceivable that positive symptoms also have a functional importance for the brain. We evaluate supporting literature data of a ‘defensive’ role of positive symptoms and the relevance of dopamine-induced neuroplasticity in the context of neurodegenerative and psychiatric diseases. Furthermore, the possible functional significance of idiopathic REMS-related behavioural disorder as well as phantom limb syndrome is examined. We suggest that positive neurological symptoms are not merely a passive expression of a damage, but active efforts, related to dopamine-induced plasticity, to maintain a correct relationship between the external world and its brain representation, thus preventing healthy cortical areas from ousting injured ones.

Central nervous system (CNS) disorders may cause extremely heterogeneous symptoms, including negative, i.e. deficit in normal functions, and positive ones, reflecting an abnormal increase in function, according to Reynolds’ distinction (1862). While the pathophysiology of the former seems rather obvious, that of the latter may not be as trivial. However, this classification exemplifies the traditional approach to CNS studies, the activities of which are interpreted from the perspective of an external observer. Indeed, evaluation of CNS alterations has usually been based on what could be perceived from the outside, which does not necessarily represent the correct interpretation of brain processes. Crucially, Buzsáki’s seminal work suggested that the CNS is not a mere tool that absorbs and encodes information from outside, but rather an explorer that formulates hypotheses and tests them to assess the correspondence between internal models and reality [1]. Importantly, his interpretation found mathematical support in the active inference theory (AIT), according to which action and perception constitute a loop that updates expectations in accordance with a Bayesian model depending on data available from the environment and existing knowledge [2]. The implications of this new interpretative model have been extensively investigated in the context of functional motor and sensory symptoms, i.e. symptoms that arise in a context of alterations in functioning of brain networks rather than abnormalities of brain structures. Indeed, functional patients not only manifest partial impairment of intentional control over the body (i.e. lack of the feeling of being the agents of their movements), but also abnormal a priori beliefs, whereby sensory evidence (bottom-up) fails to alter prior expectations (top-down) [3]. In this context, higher CNS centres must justify the emergence of wrong beliefs, as the top-down attentional processes generated the expectation, but did not predict its content, and functional symptoms emerge in an attempt to overcome the mismatch between prediction and outcome [4].

Another important theory for critically reinterpreting the implications of positive symptoms comes from an intersting explanation for the role of dreams during rapid eye movement sleep (REMS): specifically, the cerebral cortex is characterised by a continuous “conflict” between different brain areas, which constantly attempt to expand in order to acquire more of the limited available computational resources [5]. Since the human brain is mainly dedicated to visual function and visual input is deprived for several hours each day due to the sleep–wake cycle, without controlled visual “hallucinations” (i.e. dreams) the visual cortex would risk losing its territories because the other sensory modalities do not suffer such a loss during sleep. Of note, due to its high cortical neuroplasticity (i.e. the ability of the CNS to change activity in response to intrinsic or extrinsic stimuli in both physiological and pathological conditions), the visual cortex is “colonised” by tactile stimuli after just 40–60′ of visual deprivation in healthy subjects, as highlighted by a functional magnetic resonance imaging study [6]. Furthermore, a positive correlation between cortical plasticity and REMS proportion in 25 primate species was shown by Eagleman and Vaughn, who called their interpretation “Defensive Activation Theory” (DAT) in order to emphasise the defensive role played by dreams for visual areas [5].

Although highly speculative, these theories might be considered as a starting point for critically reinterpreting many concepts concerning CNS disorders from a new and intriguing perspective. For example, visual hallucinations, i.e. perceptions without an external stimulus, are common in numerous conditions, including disorders of the visual pathways, neurodegenerative (e.g. Alzheimer’s disease (AD), Parkinson’s disease (PD), Lewy body dementia (LBD)) and psychiatric diseases (e.g. schizophrenia) and delirium [7]. Albeit heterogeneous, these disorders share an alteration of the normal relationship between real objects and their brain representation, because of damage in the afferents (e.g. lesions in the visual pathway), or in the areas responsible for visual processing, due to aggregates of misfolded proteins or alterations in the balance between different neurotransmitters and neuromodulators [7]. Therefore, according to AIT, the CNS’s expectations are not fulfilled, and assuming the DAT approach, it can be hypothesised that the CNS attempts to preserve the normal functionality of visual brain areas by generating images without external stimuli, but still perceived as real by the subject. Thus, visual hallucinations are not only a symptom of visual region damage, but also perhaps an attempt by the CNS to restore the normal relationship between object and perception, which is crucial to avoid the “conquest” of these areas by other intact ones.

In particular, dopamine, which is a neurotransmitter/neuromodulator mainly produced by substantia nigra pars compacta (SNPC), has been implicated as playing a crucial role in widespread neuroplastic mechanisms [8]. Consequently, patients suffering from visual hallucinations are often treated with dopamine receptor antagonist drugs [7]: this pharmacological antagonism of dopamine makes hallucinations disappear, most likely because the neuroplasticity aimed at maintaining the correct activities of the visual regions is suppressed and the CNS loses its compensation mechanisms. In contrast, dopamine receptor agonist drugs, which may excessively enhance plasticity mechanisms and also lead to an overstimulation of the visual cortex, often cause visual hallucinations [9]. Moreover, the role of dopamine-induced plasticity for the integrity of the whole visual pathway (since it also plays a critical role for retinal cells) [10], is supported by the finding that antipsychotic agents were associated with structural impairment of the visual cortex and retina as well as an exacerbation of subjective visual perception disorders in a 3-year follow-up in patients with first-episode schizophrenia with visual disturbances [11]. Moreover, schizophrenic patients often show a dominance of the right hemisphere over the left one, characterised by alterations in frontal and temporal areas, critical for language functions. Furthermore, a correlation between abnormalities in the left arcuate fasciculus and auditory-verbal hallucinations was highlighted [12]. Thus, it is possible that structural and functional alterations in the language network of the left hemisphere force the right one to develop compensatory mechanisms, which consistently concern above all the auditory-verbal modality, leading to the typical hallucinations if predisposing factors, such as alterations in dopaminergic circuits with abnormal neuroplasticity, are present.

Regarding visual hallucinations in neurodegenerative diseases, they can occur in particular in synucleinopathies, but are not uncommon in diseases with a different pathophysiology, including AD. For example, in PD, where a progressive dopamine decline due to a degeneration of the SNPC occurs, generally visual hallucinations are experienced at a later stage of disease (i.e. when abnormal alpha-synuclein accumulation at the cortical and retinal levels is marked) or immediately after the introduction of dopamine agonists, in a dose-dependent fashion [13]. Conversely, LBD patients report characteristic visual hallucinations early on, reasonably because of the severe neuropathological alterations in the entire visual pathway, which impair visual information processing. This selectivity, interestingly, is also supported by the fact that the hallucinations of LBD patients are characterised by usually taciturn people [14]. Arguably, the CNS mainly needs to simulate inputs related to the most impaired sensory modality, whereas, for example the auditory side is less affected and, consequently, there is no need for hallucinations in order to speak. Indeed, alpha-synuclein has prion-like diffusion through the visual pathway and this could explain its preponderant and early involvement in LBD (since it is probably the starting point) and its later damage (according to Braak’s stages) in PD, justifying their different frequency and temporal sequence of visual hallucinations [15]. Furthermore, although LBD patients may also manifest parkinsonism due to dopaminergic alterations, this is not always present and is usually of lesser extent than in PD patients [14]. Thus, dopamine needed for CNS compensation is available and visual hallucinations are, consistently, predominant from the earliest stages.

Other common manifestations of synucleinopathies are sleep disturbances, often early and severe. In their prodromal phase, synucleinopathies can manifest as idiopathic REMS-related behavioural disorder (iRBD), i.e. acted dreams, excessive muscle tone and/or phasic muscle contractions during REMS, which often evolves into PD, LBD or multi-system atrophy (MSA) [16]. Importantly, iRBD patients demonstrate the same (though less severe) visual changes as PD patients (e.g. alterations of colours and stereoscopic vision, illusions), but almost exclusively PD patients present with hallucinations [17]. This different degree of severity could depend on a twofold mechanism: firstly, both conditions seem characterised by neuropathological alterations initially localised in CNS areas distant from cortical regions (e.g. brainstem) and the alpha-synuclein prion-like progression could take time, justifying the different degree of severity of the visual changes depending on the time point, since visual areas are initially spared, dopamine deficit is mild and, thus, compensation provided by visual hallucinations is not necessary at the iRBD stage[16]. Besides, despite the abnormal movements, the REMS parameters (e.g. latency, density, rate) are not substantially altered during iRBD, whereas PD is characterised by significant alterations [16]. Thus, REMS might allow the function of visual areas to be maintained in the former condition, but not in the latter, which leads to the compensatory attempt provided by visual hallucinations. This interpretation is also consistent with the different latency of symptoms between iRBD leading to PD and to LBD: since, in the latter condition, the pathology is also at the cortical level, the visual impairment is prominent and hallucinations appear early [14, 16]. In MSA, mainly characterised by a subcortical pathology, visual hallucinations are consistently very rare [18].

On the other side, abnormal movements in iRBD could be the equivalent expression in the motor areas of hallucinations in the visual pathway: since alterations in basal ganglia and brainstem circuitry compromise motor control and the dopamine decrease alters the threshold mechanism that selects motor patterns, the normal relationship between motor programme and performed action is altered [8]. According to the AIT and DAT principles and considering REMS as characterised by relevant neuroplasticity, it is possible that these movements are an attempt on the part of the CNS to restore the normal motor relations. Consistently, many movements during iRBD (particularly complex and voluntary-like ones) depend on the activation of motor regions, especially the supplementary motor area, which has direct connections with the spinal motor neurons and, therefore, bypasses the tonic inhibition that can still be partly carried out by the basal ganglia and the sub-laterodorsal tegmental nucleus (i.e. the hypothetical iRBD starting point) [16]. Lastly, iRBD patients are characterised by increased grey-matter density in the hippocampi and parahippocampal gyri. Since REMS is crucial for memory function and its physiology is altered in iRBD, this could be an attempt to enhance the activity normally performed by these regions [5, 19]. However, from the AIT and DAT perspective, as in the iRBD the relationship between input or output and its representation is altered, the CNS could try to compensate by enhancing the regions involved in mnestic functions, in order to store the correct relationships more consistently. Figure 1 summarises this information (see Fig. 1).

**Fig. 1:**
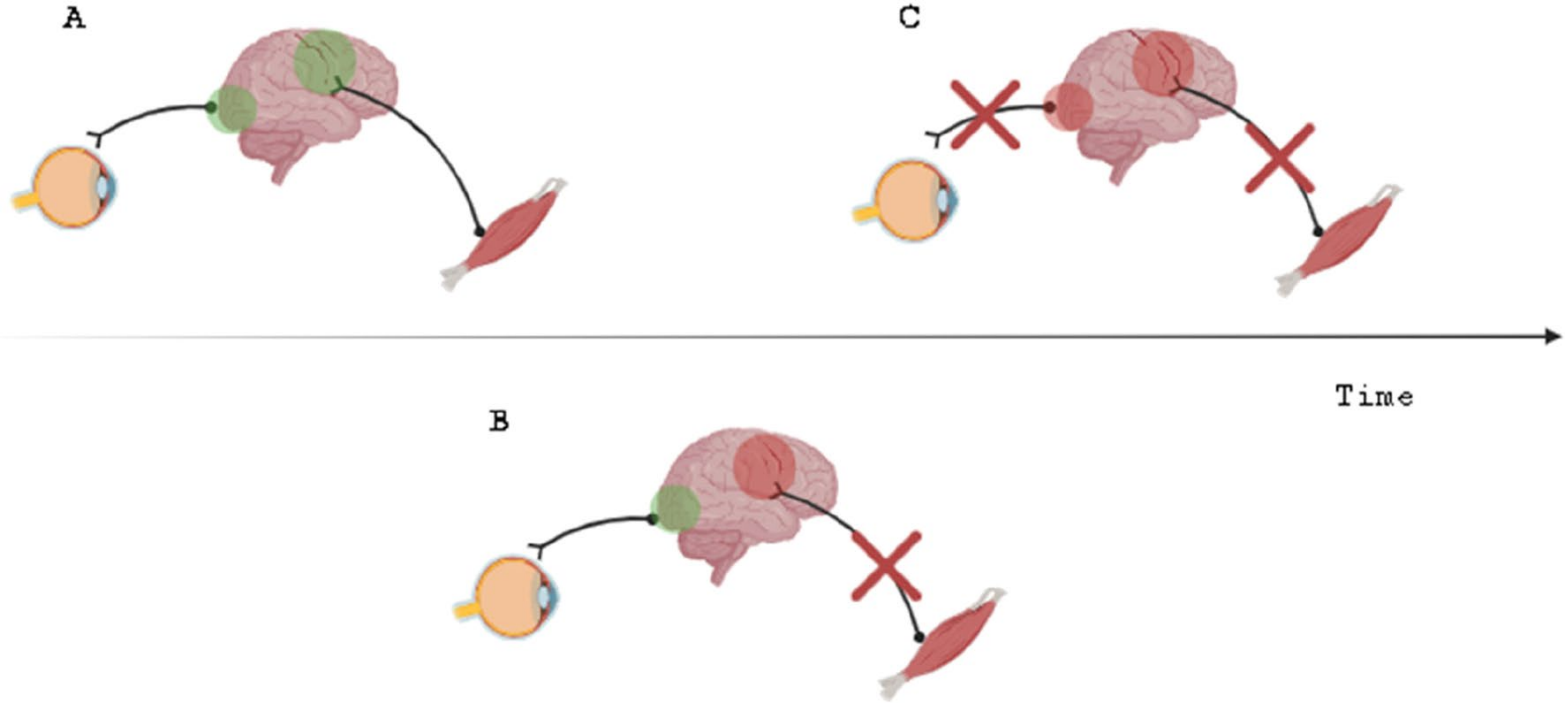
Summary of the proposed mechanism based on AIT and DAT principles in synucleinopathies. **A** Under physiological conditions, both the afferent and efferent branches of the sensorimotor loop function properly, which allows brain areas to maintain control over their cortical territory. **B** In iRBD, early damage to the motor network compromises the efferent branch of the loop; therefore, movements appear in REMS in an attempt to preserve the cortical motor territory. **C** As the neuropathological damage progresses, the parieto-occipital visual areas are impaired and the afferent branch of the loop is damaged too; therefore, similarly, visual hallucinations arise with a timing dependent on the speed and extent of the cortical damage and the availability of dopamine (i.e. LBD > PD > MSA). The arrow indicates the temporal sequence of events. Abbreviations: AIT, active inference theory; DAT, defensive activation theory; iRBD, idiopathic REMS-related behavioural disorder; *LBD*, Lewy body dementia; MSA, multi-system atrophy; PD, Parkinson’s disease; REMS, rapid eye movement sleep

Interestingly, as already mentioned, visual hallucinations can also occur in AD. In particular, although toxic Aβ oligomers mainly damage cholinergic and serotonergic neurons, recent evidence suggests that dopaminergic ones may also be vulnerable, especially in advanced stages, and the greater their involvement, the faster the cognitive decline [20]. Indeed, removing dopamine-induced neuroplastic compensation could reasonably worsen patient performance. However, it is important to note that, provided the abnormal aggregates do not affect the visual cortex, in the moderate-advanced stages, patients characteristically do not report visual hallucinations [21]. Conversely, in the posterior cortical atrophy AD variant, characterised by early alterations in the parieto-occipital regions, visual hallucinations are more frequent [21]. This suggests that the genesis of visual hallucinations is complex and multifactorial and requires structural and/or functional alterations (e.g. alterations in the balance between neurotransmitters) in the visual regions together with alterations of the neuromodulator crucial for the defensive neuroplasticity, i.e. dopamine. Finally, neurological symptoms during disorders other than neurodegeration can be analyzed within this new interpretative framework. For example, in phantom limb syndrome, characterised by the persistence of sensations (often unpleasant or painful) in body regions that are no longer present, e.g. surgically removed limbs [22], according to AIT and DAT, the cortical region representing the lost region no longer receives sensations from that body area and the CNS, therefore, creates a sensory hallucination to “defend” its cortical territory. Furthermore, unpleasant sensations elicit movements to loosen the cause of the discomfort and it is possible that the CNS uses this strategy to provoke movements in the lost part in order to force the second component of the sensorimotor loop to preserve the functionality of its cortical region. Crucially, the aforementioned interpretation fits in the hypothesis that this syndrome depends on abnormal neuroplastic mechanisms, which may play a maladaptive role in such cases [22].

This evidence could lead to the hypothesis that some CNS symptoms are not merely a passive expression of an impairment, but active attempts to maintain the correct relationships between the external world and its brain representation, which are essential to ensure that injured cortical areas continue to perform their tasks and are not displaced by other normal regions. Reasonably, dopamine, which is fundamental in neuroplastic mechanisms, could play a crucial role. To verify this interpretation, future studies should assess whether patients with more visual hallucinations have better visual function than those with fewer of them, for the same underlying neuropathological mechanism. If correct, it will allow us to use more cautiously therapies to suppress symptoms that might represent a defence mechanism of the CNS and, at the same time, offer us the mark of a critical window of opportunity to intervene before the damage becomes too pronounced and even these possible compensations lose their usefulness.

## Data Availability

Data sharing not applicable to this article as no datasets were generated or analysed. Figure created with BioRender.com.
